# On the same team: A call for increased medicolegal knowledge exchanges between forensic psychiatry and sports psychiatry

**DOI:** 10.3389/fpsyt.2022.1041891

**Published:** 2022-11-07

**Authors:** Alexander James Smith, Anna Buadze, Malte Christian Claussen, Erich Seifritz, Michael Liebrenz-Rosenstock

**Affiliations:** ^1^Department of Forensic Psychiatry, University of Bern, Bern, Switzerland; ^2^Department of Psychiatry, Psychotherapy, and Psychosomatics, Psychiatric University Hospital Zurich, University of Zurich, Zurich, Switzerland; ^3^Private Clinic Wyss AG, Münchenbuchsee, Switzerland; ^4^Adult Psychiatry, Psychiatric Services Grisons, Chur, Switzerland

**Keywords:** sports psychiatry, forensic psychiatric, medicolegal aspects, cultural competence, athlete mental health, medical malpractice, doping

## Introduction

Recently, renowned athletes have shown increasing willingness to discuss mental health. For instance, Olympic-winning gymnast, Simone Biles (1), tennis champion, Naomi Osaka (2), and cricket captain, Ben Stokes (3). Such prominent dialogues can help expand mental health literacy in competitive sports, where stigmatization represents an enduring help-seeking barrier (4). Significantly, these accounts also reflect scientific developments in sports psychiatry, an emerging interdisciplinary subspeciality and part of the broader area of sports medicine. Sports psychiatry encompasses wide-ranging expertise and clinical domains (5, 6), and has been pivotal in illuminating risk factors and mental illness rates in elite athletes (7), alongside the benefits of sports and exercise within prevention and therapeutic programmes (8). Independent societies focusing on sports psychiatry have been created [e.g., (9)] and major international associations have established dedicated sections [e.g., (10)].

Sports psychiatry shares connections with neighboring disciplines like sports psychology, clinical psychology, psychotherapy, pharmacology, and sports science, amongst others (11). However, although studies have explored sports therapy in forensic patients (12), there is scant knowledge about the medicolegal contexts of sport-specific psychopathology and its forensic-psychiatric intersections. In our view, this is concerning as elite-level competitions often transcend diverse jurisdictions, spanning legal and sporting-body regulations. For example, anti-doping offenses can entail civil or criminal cases, together with charges by regulatory bodies like the World Anti-Doping Agency (WADA) or national federations (13). Given these frameworks and sports psychiatrists' clinical responsibilities, practitioners may face complex legal questions, such as issues of litigation or medical malpractice. Equally, research underlines pertinent forensic-psychiatric trends amongst athletes, including aggressive behaviors (14), substance use disorders (SUDs) (15), and exposure to interpersonal violence and abuse (16). Consequently, should an athlete be implicated in juridical proceedings, forensic-psychiatric expertise could be required. In these circumstances, bias and sociocultural aspects in clinical practice may become more pronounced.

Due to these overlapping concerns, we believe that increased knowledge exchanges are essential between sports psychiatry, forensic psychiatry, and the law, in relation to elite-level sport. To that end, we outline possible interconnections and advocate for more epistemological interactions. In doing so, we hope to raise awareness about these medicolegal intersections, with the aim of improving psychiatric support for athletes and enhancing professional standards across these domains.

## Mental health in elite and high-professional sports

Sport-specific mental health issues are a composite clinical phenomenon. Elite athletes from different sports might be vulnerable to psychiatric morbidities (17). Albeit underexamined, symptomatology could be substantial (18), and peak onset may occur during an athlete's most competitive years (4, 15). Researchers have highlighted rates of SUDs, affective disorders, eating disorders, body dysphoria, suicidality, attention-deficit/hyperactivity disorder, and neurocognitive psychiatric deficits associated with head injuries (15, 17–20). Further, in various contexts, increased anger and aggressive behaviors have been identified (14, 21).

With their unique position in a competitive environment, elite athletes may be prone to psychopathological risk factors. Hughes and Leavey summarize these determinants (22), including overtraining, burnout, risk of injury, and sociocultural beliefs. Specifically, evidence illustrates stigmatizing paradigms about psychiatric disorders in high-performance sports (23) and these could inhibit symptom articulation (4), treatment, and help-seeking behaviors. Correspondingly, “situational narcissism” may be an exacerbating factor (24). Additional environmental and vocational stressors endure, such as performance expectations and non-support from coaches or teammates (25), and identity-based stressors, like coping with the public spotlight or fear of failure (26). Alarmingly, athletes may be susceptible to interpersonal violence and sexual and emotional abuse, influenced by environmental and sociocultural risk factors (16). Exposure to this correlates with psychopathology and might increase with athletic success, disproportionately affecting female, para, and LGBTQIA+ competitors (16).

Amidst this compound clinical environment, sports psychiatrists provide treatment for competitive athletes, delineating prevention and care pathways either independently or as part of a medical team (27). As an interprofessional area, sports psychiatry and its epistemological associations are continuously expanding, encompassing caregiving and mental health promotion (11, 28). Researchers suggest that distinctive conditions and risk factors of competitive sport could require earlier psychiatric interventions (29). Nevertheless, psychopharmacological treatment in elite-level athletes is underexamined. Concerns have been raised about this insufficient knowledge-base, notably for increases/decreases in competitive levels and athlete safety (17, 30). For example, some well-tolerated anti-depressants may need to be avoided because of potential performance limiting side-effects (31). Considerations about psychotropic substances are especially critical (32) and could lead to deviations from evidence-based guidelines (31). Stringent anti-doping regulations further aggravates these sensitivities [e.g. (33)]. Should a high-performance athlete apply for a Therapeutic Use Exemption (TUE) for a psychopharmaceutical prohibited by WADA or a national body, sports psychiatrists may be involved in this process (34).

## Sports psychiatry: Medicolegal and forensic-psychiatric intersections in competitive sports

As scientific investigations within sports psychiatry develop, there have been proposals to broaden the scope of this subdiscipline (35). Yet, to the authors' knowledge, medicolegal interconnections between sport-specific psychopathology and forensic psychiatry in competitive sports have received little scholarly attention. This lacuna is concerning as an athlete may conceivably become implicated in legal proceedings that warrant judicial responsibilities for a sports psychiatrist, and/or forensic-psychiatric expertise. This could be particularly pertinent in anti-doping infringements, with increasing calls for their criminalization [e.g. (36)]. Interestingly, some have posited that psychiatric disorders may have causality for doping, but results are scarce (37). Further, SUD rates (15) and aggressive behaviors (14, 21), alongside athletes' susceptibility to interpersonal violence and abuse (16), might also invoke cross-jurisdictional issues.

Given these medicolegal intersections, we advocate for closer collaborations between the nascent area of sports psychiatry and the forensic-psychiatric subdiscipline. Below, we outline potential scenarios, theoretical considerations, and directions for future research in competitive sporting frameworks.

### Medicolegal aspects of sports psychiatry: Legal involvement, litigation, and knowledge exchanges

Elite-level sport spans diverse civil and criminal jurisdictions, notwithstanding apposite rules enforced by regulatory bodies (38). Resultantly, sports psychiatrists may encounter complex legal questions regarding their athlete-patients and attendant medicolegal obligations. One plausible scenario is that a practitioner is treating an elite-level athlete who resides in one jurisdiction but is facing/pursuing litigation in another. Amongst other responsibilities, a sports psychiatrist might be subpoenaed to transfer medical data to legal actors or to a forensic-psychiatric expert as collateral information; for instance, in cases of interpersonal violence, causality may need to be established within psychopathological contexts. In these circumstances, concerns would materialize about testimony, confidentiality and patient privacy, and informed consent. These are all conditioned by specific judicial regulations (39). Additionally, exposure to protracted legal settings could complicate mental health support for elite-level athletes, heightening stressors and affecting treatment; sports psychiatrists need to be aware of these possibilities. Studies show that defendants and litigants adopt poor coping strategies and exhibit increased psychosomatic symptoms (40, 41). These may be prevalent in lengthy and complicated anti-doping proceedings, particularly since certain situations will involve criminal charges (36), or adjudication by the Court of Sporting Arbitration. Future studies specifically focusing on the stressors of anti-doping charges would inform appropriate psychiatric support.

Owing to their potential involvement with TUE processes, prescribing psychotropic medications, and providing medical advice, sports psychiatrists might face ethical and legal issues, either through deliberate actions or inadvertent negligence. As an example, a sports psychiatrist may be caring for an athlete-patient who is traveling between different international sporting events. This could mean that they are asked to prescribe psychopharmaceuticals in jurisdictions where they are not licensed to practice. Alongside inherent legal risks and ethical concerns, this may increase the possibilities of an athlete self-medicating, creating higher chances of doping contraventions and adversely affecting patient safety. Alternatively, this could also lead to treatment provision by non-psychiatric specialists, again jeopardizing an athlete's welfare. Furthermore, Landis has explicated numerous situations that can necessitate juridical interactions for sports physicians (42). In notorious basketball doping breaches, researchers suggested that clinicians failed in their duty of care and exhibited poor judgement (43). Given potential deviations from accepted therapeutic guidelines may occur (31), and with increasing mental health awareness and treatment requirements in elite competitions, sports psychiatrists may be more exposed than other psychiatric subspecialties to litigation.

Whilst sports psychiatrists must have sufficient expertise about anti-doping rules, WADA and governance bodies continuously (and separately) update them, causing inconsistencies. For instance, at the time of writing, the opioid, Tramadol, is prohibited in-competition by the international cycling federation, the Union Cycliste Internationale, but not by WADA (44) (Tramadol is on WADA's Monitored List but not its Prohibited List). As doping offenses often result in career-ending charges, sports psychiatrists could face legal proceedings initiated by their athlete-patients for negligence or malpractice. The WADA code comprises “strict liability,” stipulating sole athlete responsibility for substance use. Yet, the legal basis of this has been previously criticized [e.g., (45)] and further intricacies arise if medical malpractice or negligence are proven; how would these aspects be appraised in civil or criminal jurisdictions? Similarly, complexities remain about the procedures of general medical licensing bodies in these situations [e.g., (46)]. In our view, such issues need detailed ethical and legal consideration, balancing notions of physician and patient responsibility in sporting domains.

Navigating legal scenarios could require extensive judicial insights, which might be lacking in the developing subspeciality of sports psychiatry. With this in mind, additional training about legal conventions and forensic psychiatry will prove beneficial to sports psychiatry, like in general psychiatry (47). Recently, discussions about educational programmes have occurred and we applaud the formulation of robust curricula [e.g., (48–50)]. However, in our opinion, these initiatives should incorporate jurisdictional aspects to bolster professional and clinical expertise. Moreover, the dissemination of consensus papers on medicolegal topics would further progress these knowledge exchanges, as would evidence-based practice guidelines (51). In this regard, professional societies could help facilitate interdisciplinary dialogues, especially as dedicated associations and specific sections for sports psychiatry are being established at an organizational level (9, 10).

### Elite-level sport and forensic psychiatry: Evaluations, sociocultural considerations, and potential biases

Across diverse legal settings, a forensic psychiatrist might encounter an elite-level athlete in the context of criminal and civil matters, like in cases of interpersonal violence, substance use and doping-related offenses, or during medical malpractice complaints, as outlined.

Specifically, medical malpractice constitutes a compound issue and presumes that a sports psychiatrist has demonstrated non-compliance with recognized professional standards in the therapeutic relationship, thus requiring forensic-psychiatric expertise (52). Causation would need to be established; there must be reasonable correlations between the psychiatrist's acts and any damages experienced by the athlete (42, 53). Conceptual problems could arise in situations where deviations from treatment guidelines have transpired because of performance limiting side-effects. Here, the notion of patient informed consent might be critical (54) and could shape subsequent legal proceedings. If therapeutic choices were based on possible adverse performance effects rather than individual treatment needs, this could implicate additional stakeholders who may also have influenced these decisions, like other members of the athlete's medical team, support staff, or their coaches.

To uphold best practice, forensic psychiatrists must be aware of the unique risk factors of high-performance competitors. For example, Bär and Markser have ascribed a specificity to sporting mental health, which may influence etiology and clinical presentation (55). Likewise, some propose shifting diagnostic approaches to sport-specific mental disorders, from a general normal-versus-pathological to a tailored functional-versus-dysfunctional model (56). The validity of this dialectic in forensic-psychiatric assessments goes beyond the scope of our discussions and could form the basis for future research. Nevertheless, as highlighted, elite-level athletes come from a composite milieu where mental illnesses may be deliberately minimized and stigmatized. Therefore, during forensic-psychiatric evaluations, dissimulation could occur (the downplaying or concealment of symptoms by the evaluee). Forensic psychiatrists might need to be vigilant for this when assessing professional athletes; symptom articulation and diagnoses could be affected. Despite empirical validation amongst the general population and not within this demographic, psychometric tests can help uphold diagnostic objectivity and mitigate against these behaviors (57, 58).

Amidst these frameworks, cultural competency in forensic-psychiatric practice would assume greater importance. Researchers argue that cultural and socioeconomic determinants can help contextualize psychopathology and inform legal outcomes (59). Accordingly, forensic psychiatrists should consider these concepts in relation to competitive sports; cultural bridges would ensure holistic and well-informed opinions. Again, learnings could be taken from sports psychiatry, which underscores the sociocultural dimensions of elite-level athlete's mental health. Consulting other experts with advanced knowledge about professional athletes, including sports psychiatrists, psychologists, and general physicians, may be beneficial. Moreover, cultural brokers can fulfill a valuable role and have shown efficacy elsewhere in forensic-psychiatric activities (59).

Possible biases might be introduced by forensic psychiatrists toward athletes. Alongside others, the anchoring effect could shape decision-making processes given prominent media narratives about athletes in the public eye. Notably, this may be pertinent in anti-doping incidents, which can provoke emotive press reports (60). Other concerns center around dual agency bias, particularly if the forensic mental health expert has previously interacted with the athlete or their medical team (61). Forensic psychiatrists need to be mindful of these possibilities and employ appropriate bias mitigation strategies.

## Concluding remarks

Sports psychiatry is a burgeoning subspecialty with multidisciplinary implications for elite-level sports. However, there is scant awareness about its jurisdictional intersections with forensic psychiatry and the law. We sought to bridge this gap by examining prospective overlaps and presenting broad questions, concerns, and potential research directions in elite and high-performance sports. Consequently, we hope this forms the basis for increased knowledge exchanges, with the aim of raising awareness about medicolegal issues, enhancing professional standards, and optimizing cross-disciplinary psychiatric support.

## Author contributions

AS, AB, and ML-R conceptualized this paper. AS and ML-R wrote the first draft of the paper. All authors contributed to manuscript edits and provided feedback for subsequent drafts and read and approved the submitted version of this manuscript.

## Conflict of interest

The authors declare that the research was conducted in the absence of any commercial or financial relationships that could be construed as a potential conflict of interest.

## Publisher's note

All claims expressed in this article are solely those of the authors and do not necessarily represent those of their affiliated organizations, or those of the publisher, the editors and the reviewers. Any product that may be evaluated in this article, or claim that may be made by its manufacturer, is not guaranteed or endorsed by the publisher.
